# Evaluation of Infection Prevention and Control Readiness at Frontline Health Care Facilities in High-Risk Districts Bordering Ebola Virus Disease–Affected Areas in the Democratic Republic of the Congo — Uganda, 2018

**DOI:** 10.15585/mmwr.mm6839a4

**Published:** 2019-10-04

**Authors:** Caitlin Biedron, Meghan Lyman, Matthew J. Stuckey, Jaco Homsy, Mohammed Lamorde, Ulzii-Orshikh Luvsansharav, Kathryn Wilson, Danica Gomes, Winifred Omuut, Solome Okware, Judith Nanyondo Semanda, Reuben Kiggundu, Daniel Bulwadda, Vance Brown, Lisa J. Nelson, Alfred Driwale, Ryan Fagan, Benjamin J. Park, Rachel M. Smith

**Affiliations:** ^1^Epidemic Intelligence Service, CDC; ^2^Division of Healthcare Quality Promotion, National Center for Emerging and Zoonotic Infectious Diseases, CDC; ^3^CDC Uganda, Center for Global Health, CDC; ^4^Infectious Diseases Institute, Makerere University College of Health Sciences, Kampala, Uganda; ^5^Uganda Ministry of Health, Clinical Services, Kampala, Uganda

Infection prevention and control (IPC) in health care facilities is essential to protecting patients, visitors, and health care personnel from the spread of infectious diseases, including Ebola virus disease (Ebola). Patients with suspected Ebola are typically referred to specialized Ebola treatment units (ETUs), which have strict isolation and IPC protocols, for testing and treatment (*1*,*2*). However, in settings where contact tracing is inadequate, Ebola patients might first seek care at general health care facilities, which often have insufficient IPC capacity (*3*–*6*). Before 2014–2016, most Ebola outbreaks occurred in rural or nonurban communities, and the role of health care facilities as amplification points, while recognized, was limited (*7*,*8*). In contrast to these earlier outbreaks, the 2014–2016 West Africa Ebola outbreak occurred in densely populated urban areas where access to health care facilities was better, but contact tracing was generally inadequate (*8*). Patients with unrecognized Ebola who sought care at health care facilities with inadequate IPC initiated multiple chains of transmission, which amplified the epidemic to an extent not seen in previous Ebola outbreaks (*3*–*5*,*7*). Implementation of robust IPC practices in general health care facilities was critical to ending health care–associated transmission (*8*). In August 2018, when an Ebola outbreak was recognized in the Democratic Republic of the Congo (DRC), neighboring countries began preparing for possible introduction of Ebola, with a focus on IPC. Baseline IPC assessments conducted in frontline health care facilities in high-risk districts in Uganda found IPC gaps in screening, isolation, and notification. Based on findings, additional funds were provided for IPC, a training curriculum was developed, and other corrective actions were taken. Ebola preparedness efforts should include activities to ensure that frontline health care facilities have the IPC capacity to rapidly identify suspected Ebola cases and refer such patients for treatment to protect patients, staff members, and visitors.

The Ebola outbreak in DRC was declared on August 1, 2018. As of September 22, 2019, a total of 3,168 probable and laboratory-confirmed cases had been reported in the outbreak, 3,162 (99%) of which were reported from North Kivu (Nord-Kivu) and Ituri provinces, in the northeastern part of the country, bordering Uganda (*9*). Six additional cases have been reported from South Kivu (Sud-Kivu), which borders Rwanda and Burundi (*9*). Health care personnel have accounted for 160 (5%) cases (*9*). Cases initially were confirmed in Mandima health zone in Ituri province, but the epicenter of the outbreak subsequently moved southward through North Kivu, to the Beni, Katwa, and Butembo health zones, where the majority of cases are currently being reported (*9*). Cases continue to be identified across a large swath of territory spanning Ituri, North Kivu and South Kivu provinces, and outbreak control has been hampered by population mobility, insecurity, and community mistrust of response activities. Official and unofficial cross-border movement between Ituri and North Kivu provinces and Uganda occurs for trade, family visitation, movement of refugees, and medical care, increasing the risk for importation of Ebola into Uganda.

In August 2018, baseline IPC assessments were performed with a convenience sample of four health care facilities in Uganda selected because of their proximity to the focus of the Ebola outbreak in DRC. Institutional review board review was not performed for this activity because the IPC assessments were part of a public health program evaluation in an emergency response. The facilities included one regional referral hospital, two district hospitals, and one Level IV health center. Assessment teams included staff members from district health offices, Makerere University’s Infectious Disease Institute (IDI), and U.S. CDC. Upon arrival at the facility, assessment teams first met with the medical director to explain the assessment. Interviews, using a semistructured questionnaire,* were then conducted with the frontline health care personnel (including the IPC nurse-in-charge or main IPC focal point for the facility, physicians, nurses, and environmental cleaners) responsible for conducting screening, isolation, and notification procedures. The assessments also included examination of the facility and observation of practices and focused on a facility’s readiness to prevent Ebola transmission. Capacity in three major domains was assessed: 1) safe and systematic screening and identification of patients with signs and symptoms of Ebola; 2) isolation of any patient meeting the case definition for suspected Ebola; and 3) reporting of patients with suspected Ebola to the required public health authorities. Other general IPC practices were also assessed, including hand hygiene, proper use of personal protective equipment (PPE), and waste disposal. The assessment tool comprised a list of questions within each of the major domains and included observations of current facility isolation and screening practices, if possible. Additional open-ended questions were included to probe further into findings identified in the structured portion of the questionnaire.

Within the screening domain, the assessment focused on determining the location of the screening station, assessing availability of screening supplies, reviewing social distancing practices and use of a standardized case definition, and assessing the capacity of screening staff members. The assessment of isolation focused on ascertaining the availability of IPC consumables and other supplies, reviewing the suitability of the isolation area layout and the designated PPE donning and doffing areas, assessing whether the chlorine dilution process was performed properly, reviewing appropriate waste disposal, and assessing the level of training of health care personnel caring for isolated patients. Within the notification domain, the assessment focused on whether staff members were aware of the proper public health authority to contact when a suspected case was identified, whether a posted list of contact numbers for the district health office was available, and whether a functional mobile phone with adequate phone credit had been provided to staff members.

The assessments were conducted at facilities in Bundibugyo, Kabarole, and Kasese districts, in western Uganda (Figure). Assessment results indicated that IPC preparedness was lacking in several important areas within each of the three domains (Table). Safe and systematic screening was hindered by use of multiple case definitions, improper use of infrared thermometers, and poor adherence to social distancing measures when screening patients. Facility isolation capacity was affected by shortages of IPC consumables such as PPE, training gaps among staff members, and absence of a clear case management and referral plan (i.e., how suspected patients would move from frontline facilities to ETUs). In some facilities, isolation areas were currently in use and several deviations from best practices were seen, including patients with suspected Ebola being unattended, improper chlorine dilution, and improper disposal of PPE and other waste. The assessment team also noted that several of the facilities were in the process of building structures intended to become ETUs; however, these facilities did not have functional isolation areas for suspected Ebola patients who might come to the facility for general health care while the ETUs were still in the process of being built. Similarly, training for health care personnel was primarily focused on ETU-related IPC and case management and not on recommended screening and isolation procedures for general health care facilities. In terms of notification practices, most staff members were aware that a district rapid response team existed; however, they had not been informed of which number to call if a suspected Ebola case was identified and contact numbers for the district health office were not clearly posted.

**FIGURE:**
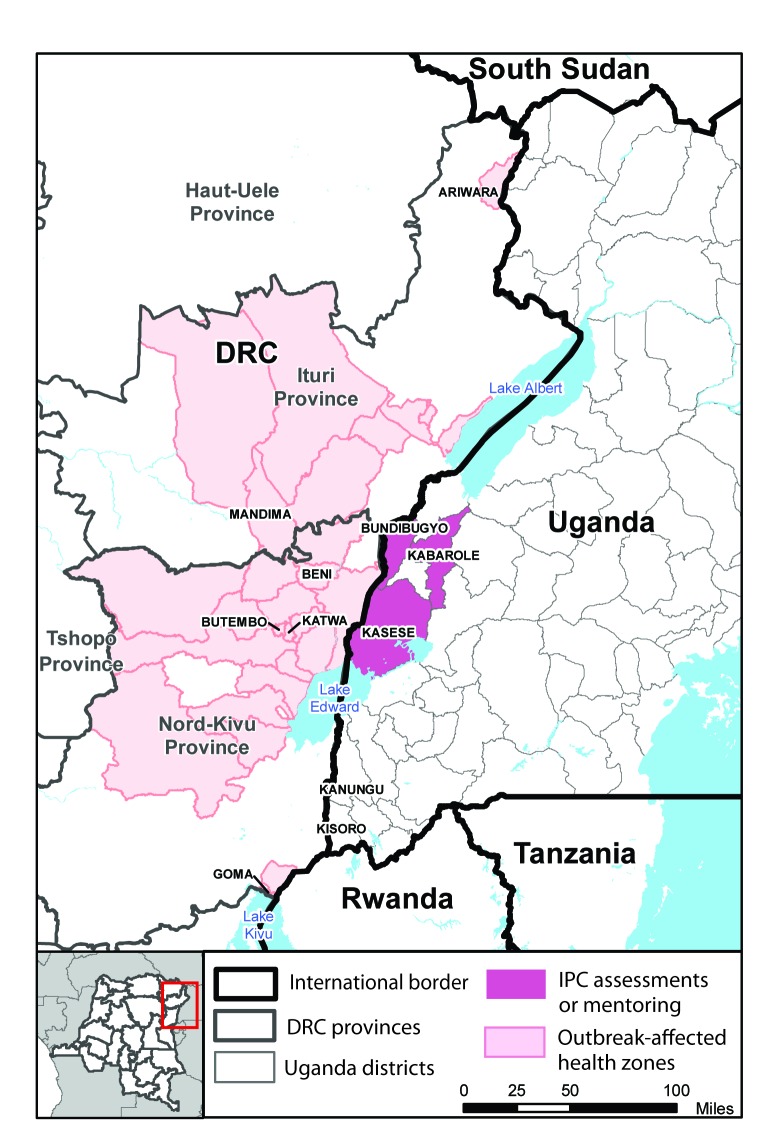
Location of Ebola virus disease outbreaks and frontline health care facilities conducting baseline infection prevention and control (IPC) assessments — Democratic Republic of the Congo (DRC)–Uganda border region, 2018

**TABLE:** Infection prevention and control (IPC) evaluation domains assessed and gaps identified in four health care facilities — Bundibugyo, Kabarole, and Kasese districts, Uganda, August, 2018

Components assessed	Gaps identified
**Screening**	
Location of screening station	—*
Availability and proper use of supplies	Improper use of infrared thermometers
Social distancing practices^†^	Poor adherence to social distancing measures
Use of a standardized case definition	Use of multiple case definitions
Staff member capacity	Gaps in training
**Isolation**	
Availability and proper use of supplies^§^	Shortage of PPE
Suitability of layout	Lack of functional isolation areas for persons seeking general health care; unattended patients with suspected Ebola
PPE donning and doffing areas	—*
Quality of chlorine preparation	Improper chlorine dilution
Waste disposal	Improper PPE and waste disposal
Staff member training	Absence of clear case management plan
**Notification**	
Knowledge of how to contact public health authority	Staff members not informed of number to call when a suspected case is identified
Availability of posted contact numbers	Contact numbers for district health officers not posted
Availability of functional mobile phone	—*
Adequate phone credit	—*

**Abbreviation**: ETU = Ebola virus disease treatment unit; PPE = personal protective equipment.

* No gaps identified.

^†^ Social distancing refers to maintaining a proper distance (usually recommended to be 1–2 m) between persons (e.g., the health care provider and the patient being screened).

^§^ IPC supplies include infrared thermometers, PPE (gloves, mask, gown, and shoe coverings), supplies for hand-washing station (water, soap, and paper towels), chlorine, plastic container with lid for chlorine, waste bins, bin liners, and sharps boxes.

## Discussion

A summary of the baseline IPC assessment findings was presented during the Ebola National Task Force meeting held on August 22, 2018, to Uganda Ministry of Health (MOH) staff members and other stakeholders present at the meeting. Based on the findings, the National Task Force identified additional funds to purchase needed IPC supplies. Furthermore, the Uganda MOH, CDC, and Makerere University’s IDI developed a training curriculum targeting the identified IPC weaknesses and a strategy to provide IPC mentorship to priority health care facilities within high-risk districts. An initial training of 23 national and district mentors was conducted on September 12, 2018, focused on screening, isolation, and notification of patients with suspected Ebola and other IPC topics. The national mentors who attended the training included representatives from the Uganda MOH, staff members from IDI, and clinicians from other district hospitals who had received previous IPC training. District health officers from a subset of high-risk districts also participated in the training. Mentorship teams that included one national mentor and one district mentor were created. Mentors have begun performing on-site mentorship at priority facilities to set up screening and isolation areas and to ensure that facilities are conducting appropriate screening, isolation, and notification. Training materials and curricula have been shared with partners in Rwanda and South Sudan to strengthen Ebola IPC preparedness in other countries neighboring DRC. In addition, this preparedness work is consistent with the capabilities that Uganda has been building under the Global Health Security Agenda and the International Health Regulations framework.

The southward spread of confirmed Ebola cases in late 2018 to the Butembo and Katwa health zones of DRC identified additional high-risk districts in Uganda; trainings of mentors and health care personnel have now been conducted in Kanungu and Kisoro districts. On June 11, 2019 the Uganda MOH confirmed the initial cluster of three Ebola cases in Kasese district (*10*). One additional Ebola case was confirmed shortly after identification of this initial cluster (*9*). Subsequently, an additional round of training for 25 mentors in the Kasese district was led by IDI and scaled up to cover 117 facilities with a goal of reinforcing IPC preparedness and improving practice. As of September 27, 2019, no additional Ebola cases have been identified in Uganda, but the extension of the outbreak into Uganda underscores the need to maintain high levels of IPC preparedness throughout districts bordering affected health zones in DRC.

The findings in this report are subject to at least two limitations. First, only four facilities were assessed during this evaluation and a convenience sample was used. Given the limited sample size and that facilities were not randomly selected, the findings might not be representative of the IPC practices at other health care facilities in the region and might not be generalizable. Second, not all facilities were actively isolating patients with suspected Ebola at the time of the assessment; therefore, certain IPC practices could not be observed. However, at such sites, the staff members were asked how they would perform certain IPC activities if a suspected Ebola patient were to be admitted.

Ebola outbreaks necessitate rapid scale-up of IPC preparedness activities at facilities where the risk for encountering patients with Ebola is high. Although planning for the establishment of well-run, functional ETUs is a critical aspect of Ebola preparedness, IPC readiness at frontline general health care facilities is also critical to preventing the spread of disease and propagation of outbreaks. Recognition of this necessity in Uganda led to the rapid development and implementation of a plan to enable general health care facilities to promptly identify patients with suspected Ebola and refer them for appropriate management. Close collaboration between the Uganda MOH and district health offices has also been critical, and ongoing engagement of district health officers will be needed for coordination of local mentorship activities and sustainability of IPC preparedness efforts.

**Summary**

**What is already known about this topic?**

The 2014–2016 West Africa Ebola virus disease (Ebola) outbreak demonstrated the importance of strengthening infection prevention and control (IPC) capacity at frontline health care facilities to prevent health care–associated transmission.

**What is added by this report?**

IPC assessments were performed in four frontline health care facilities in Uganda shortly after an Ebola outbreak in neighboring Democratic Republic of the Congo was recognized. Recommendations were made to address identified gaps in screening, isolation, and notification practices.

**What are the implications for public health practice?**

Ebola preparedness should include a focus on ensuring that general health care facilities have the capacity to rapidly identify suspected Ebola cases and refer patients for treatment to protect patients, staff members, and visitors.

## Footnotes

* The IPC assessment questionnaire used during this activity was modified from assessment tools previously used by CDC staff members during the West Africa Ebola outbreak. The questionnaire has not been formally validated or piloted. Engagement and informal interviews with health care workers occurred during the assessment process to determine if the facility’s capacity in each of the domains was adequate.
